# 

**Published:** 1896-06-27

**Authors:** 


**Notices of Births, Deaths, and Marriages** are inserted in this column at a charge of 2s. 6d. for an announcement not exceeding four lines.

**Notice to Advertisers and Subscribers.**
All Advertisements, Orders for Copies of The Hospital and Business Communications should be addressed to **THE MANAGER, (Not to The Editor),** The Hospital, 428, Strand, London, W.C.

The Cost of Subscription, post free, is as follows:—Per week, 2½d.; per quarter, 2s. 8d.; per half-year, 5s. 3d.; per annum, 10s. 6d. Foreign:—Per Annum, 15s. 2d.; payable, in all cases, in advance.

Just Published.
**"Burdett's Hospitals and Charities, 1896."**

Seventh year of publication. Over 1,000 pp. Scarlet cloth, gilt lettered, Price 5s. A most complete guide to British, Colonial, Indian, and American Hospitals, Dispensaries, Nursing and Convalescent Institutions, and Asylums. A few complete sets of the preceding six issues of the "Annual" may still be obtained on application to the Publishers, The Scientific Press (Limited), 428, Strand, London, W.C.

### NOTICE TO CORRESPONDENTS.

All MS., Letters, Books for Review, and other matters intended for the Editor should be addressed The Editor, The Lodge, Porchester Square, London, W.

The Editor cannot undertake to return rejected MS., even when accompanied by stamped directed envelope.

**NOTICE.—Vol. XIX. of "The Hospital" (October, 1895, to April, 1896), in handsome cloth binding, is now on sale at the Office of this Journal. Price 7s. 6d. Cases for binding the Numbers contained in this Volume, 1s. 6d. Index to Vol. XIX., 2½d., post free. The Monthly Part for July will be ready on July 1st, price 6d., by post 8d.**

### SCRAPS AND GLEANINGS.

The Belfast Hospital Saturday collection was made on the 30th ult.

Miss Ann Daniel has left a legacy of £1,000 to the Missions to Seamen.

The London City Mission has now 481 missionaries engaged in work in the metropolis.

A proposal to establish a dental hospital for Nottingham is at the present moment "in the air."

The Belfast Hospital for Consumpton is to be amalgamated with the Forster Green Hospital for Consumption.

It is satisfactory to note that the ladies of Hastings are about to establish a system of house-to-house collections in aid of the hospital.

It is intended in future, if possible, to hold the Hospital Saturday collection for all the Wolverhampton hospitals on the same day, instead of on two days, the present custom.

Dr. E. C. Hope, of Geelong and Melbourne, obtained on Friday last his diplomas of M.R.C.S. and L.R.C.P., London. Dr. Hope is proceeding to the colony of Victoria next month.

Out of a total income in 1895 of £967 the members of the Leamington Provident Dispensary supplied no less than £851. 5,277 cases were attended requiring 30,000 consultations.

Forty-six medical and 74 surgical cases were treated during the year ended March 1st, 1896, at the Falkirk Cottage Hospital. Of these nine died, making a death-rate of 7·5 per cent.

We are glad to see that the Cleveland Cottage Hospital is falling into line with other institutions, and has decided to make up its accounts to December 31st, instead of to September 30th, each year.

An average of 32 fresh patients were treated every week at the Bristol Eye Dispensary during 1895, giving an average attendance of double that number. 1,667 patients were treated during the year.

The Sunderland Board of Guardians have decided that the plans for the new workhouse hospital, which they had accepted, are too costly. They have therefore to referred the matter back to their Buildings Committee.

At the annual meeting of the Devonshire Hospital and Buxton Bath Charity it was stated that 2,834 in-patients had been treated during the year ended April, 1896. No less than 2,389 of the cases treated were of an arthritic rheumatic or gouty character.

Sir Benjamin Ward Richardson, in the course of an interview the other day, stated that alcohol had only been administered 19 times at the London Temperance Hospital since 1873, although over 12,000 in-patients had been treated during that time.

The annual report of the Retford General Dispensary and Cottage Hospital states that a legacy of £100 was received during the year 1895 6, and gifts of £300 and £100, the latter sum towards building a larger room for operations. This operation room is now practically completed.

In spite of the fact that scarlet fever was still rife in the town, the General Committee of the Stockton Surgical Hospital, by ten votes to eight, rescinded the rule allowing only adults to visit patients which was passed with the object of preventing the introduction of the disease into the hospital.

The in-patients treated at the Kent County Ophthalmic Hospital in 1895 have increased, when compared with 1894, from 334 to 364, and the out-patients from 2,8?8 to 2,866. The expenditure shows a slight decrease, viz., from £1,548 to £1,493, chiefly owing to the cost of repairs being less in 1895 than in 1894.

Through the energetic action of the committee appointed by the parishioners of Marnoch and Forglen, N.B., that portion of the bequest left by Miss Rose Innes for the endowment of the Aberchider Cottage Hospital, which had been misappropriated by the trustees' late law agent, has been made good by the trustees. The whole amount with interest (in all £2,877) has been paid to the hospital authorities.

The new Eye, Ear, and Throat Hospital for Londonderry and the North-West District was opened on the 28th April. The new hospital stands on the site of some half-dozen old houses in Bridge Street, Londonderry, which, with about a dozen others, at one time formed what was known as "Ann's Close." The hospital contains room for 12 in-patients.

The rule confining the use of the Ashby-de-la-Zouch Cottage Hospital to the district within a six miles' radius from the parish church of that town has now been altered, and this will no doubt be a gain to the hospital in the matter of subscriptions from persons residing outside the magic circle. Patients can now be received from "Ashby-de-la-Zouch or neighbourhood."

The proposal to convert the old Hebburn Hall into an accident hospital for the Hebburn district has been enthusiastically received. The need for a hospital in this neighbourhood will be evident when we state that there are in the district twelve works and factories employing between 5,000 and 6,000 workmen, and also that in four of these works alone there were taking an average of the accidents during the years 1892 to 1895, 500 accident cases per annum.

The enlargement and improvement of the Coventry Provident Dispensary has absorbed the balance of the reserve fund and left a debt of nearly £300. Instead of making a special appeal for this sum, the committee have pluckily decided to pay this amount out of the ordinary receipts of the fund. The total income for the year was as follows: Free fund, £2,861; honorary fund, £64; reserve fund, £237; and the expenditure—free fund, £1,718; honorary fund, £1,033; reserve fund, £247.

The Board of Guardians of the Ballinasloe Union have adopted a resolution calling on the Government to repeal or amend the Galway Hospital Act, with a view of relieving the unions in county Galway of what they describe as "a most unreasonable and oppressive tax." The resolution sets out that the Galway Hospital "costs the Galway portion of the Ballinasloe Union the sum of £136 16s. 3d. annually, besides paying 1s. a day for each patient sent, for which we receive no equivalent benefits; that patients sent to Dublin hospitals, under similar circumstances as we have sent them to the Galway Hospital, would cost very little more for maintenance without the enormous annual sum we have to pay to the Galway Hospital. This shows the great hardship perpetrated on the county Galway unions."

### Books Received.

The Scientific Press (Limited).

"Clinical Diagnosis." By W. G. Aitchison Robertson.

"Burdett's Hospitals and Charities, 1896." By Henry C. Burdett.

Sampson Low, Marston and Co.

"20th Century Practice of Medicine." Edited by T. L. Stedman, M.D. Vol. V., "Diseases of the Skin."

J. and A. Churchill.

"The Pathology of the Contracted Granular Kidney, and the Associated Cardio-Arterial Changes." By Sir G. Johnson, M.D., F.R.C.P., F.R.S.

"A Manual of Botany." By J. Reynolds Green. Vol. II.

"The London Manual, 1896-7." Published at the offices of "London," 125, Fleet Street.

Smith, Elder, and Co.

"The Spas and Mineral Waters of Europe." By Hermann Weber, M.D., and F. Parkes Weber, M.D.

A. and C. Black.

"Strathpeffer Spa." By R. Fortescue Fox, M.D.

Kegan Paul, Trench, Trubner, and Co.

"B. Bradshaw's Dictionary of Bathing Places and Climatic Health Resorts."

J. Lebigue et Cie.

"A New Treatment of the So-called Incurably Deaf People." By Julian J. Hovent, M.D.

T. Burleigh.

"State Aided v. Voluntary Hospitals." By W. Knowsley Sibley, M.D.

The "Family Doctor" Publishing Company.

"Girlhood and Wifehood." By a Surgeon and Accoucheur.

Whittaker and Co.

"Modern Optical Instruments." By Henry Orford.

**Periodicals and Pamphlets.**—Zenana, Humanitarian, English Illustrated Magazine, Literary Digest, Medical Week, Medical Pioneer, Clinical Journal, Cornhill Magazine, Australasian Medical Gazette, Columbus Medical Journal, New York Medical Journal, London, Cassell's Natural History, Nursing Notes, Cassell's Family Magazine, Electrical Review, Leeds Hospital Magazine, Invention, Pediatrics, Medical Press, Sanitary Journal, Practitioner, Tri-State Medical Journal, Indian Medico-Chirurgical Review, Erin, Monthly Homœopathic Review, Charities' Review, Medical Bulletin, Inter-colonial Medical Journal of Australasia, Westminster Review, Charlotte Medical Journal, Columbus Medical Journal, Herald of Health, Guy's Hospital Gazette, International Medical Magazine.

### The Student of Medicine.

### APPOINTMENTS.

J. Anderson, M.B.Glasg., L.R.C.S.Edin., appointed Medical Officer for the Poulton District of the Fylde Union. Mr. A. J. Atkins, appointed Assistant Medical Officer and Dispenser at the Lambeth Union Workhouse. J. C. Baker, appointed Medical Officer to the Workhouse of the Hambledon Union, and Surgeon to the Foresters' Court "Pinewood." P. T. B. Beale, F.R.C.S.Eng., appointed Examiner in Elementary Biology for for the First Examination of the Royal College of Surgeons of England. J. R. Bradford, M.D.Lond., M.R C.S.Eng., appointed Examiner in Elementary Physiology for the First Examination and Examiner in Physiology for the Second Examination of the Royal College of Surgeons of England. J. M. Bryan, M.R C.S., L.R.C.P.Lond., appointed Medical Officer of Health for the St. James's (Northampton) Urban Sanitary District. G. A. Buckmaster, M.A., M.D.Oxon., M.R.C.S.Eng., appointed Examiner in Elementary Biology for the First Examination of the Royal College of Surgeons of England. Miss M. M. T. Christie, M.B.Lond., appointed Assistant Medical Officer to the Greenwich Union Workhouse. H. S. Collier, F.R.C.S., appointed Surgeon to Out-patients, Paddington Green Children's Hospital. A. W. Collins, M.B.Vict., L.R.C.P.Lond., appointed Medical Officer to Ulverston Union Workhouse. G. Cowie, M.B., appointed Assistant Medical Officer to the Whitechapel Union Infirmary. A. Davidson, M.B., C.M.Aberd., appointed Senior Assistant Medical Officer to the County Asylum, Dorchester. W. Duncan, M.D., F.R.C.S.Eng., M.R.C.P.Lond., appointed Examiner in Midwifery for the Third Examination of the Royal College of Surgeons of England. P. Galloway, M.B., M.S.Aberd., appointed Medical Officer for the Willingham District of the Gainsborough Union. Dr. G. Giffen, appointed Medical Officer to the Tarbin Union Workhouse. R. Green, M.D, B.Hy.Durh., appointed Medical Officer of Health to the County Borough of Gateshead. M. Handfield-Jones, M.D.Lond., M.R.C.S.Eng., appointed Examiner in Midwifery for the Third Examination of the Royal College of Surgeons of England. W. F. Haslam, F.R.C.S.Eng., appointed Examiner in Anatomy for the Fellowship of the Royal College of Surgeons of England. H. Head, M.A., M.D.Camb., M.R.C.P.Lond., M.R C.S.Eng., appointed Medical Registrar to the London Hospital. H. J. Hibberd, M.R.C.S.Eng., L.S.A., appointed Medical Officer for the Brockenhurst District of the Lymington Union.

### VACANCIES.

The following vacancies are announced this week, the amount of salary in each case being given after the name of the institution:—

### Resident Medical Officers.

Brighton and Hove Lying-in Institution and Hospital for Women.—House Surgeon; unmarried, and when elected under 30 years of age. Salary, £80 per annum, with furnished quarters and board, coals, gas, and attendance. Applications to the Honorary Secretary, 76, West Street, Brighton, before July 1st.

Central London Ophthalmic Hospital, 238a, Gray's Inn Road. W.C.—House Surgeon. Applications to the Secretary before July 7th.

Chester General Infirmary.—Visiting Surgeon; must be doubly qualified. Appointment for not more than two years. Salary to commence at £90 per annum, with board and residence. Applications to the Chairman of the Board of Management, Secretary's Office, 29, Eastgate Row, North Chester, by June 27th.

General Hospital, Barbadoes.—Junior Resident Surgeon. Appointment for three years. Salary £200 per annum, payable monthly, with unfurnished quarters. Passage paid. Applications to the Secretary, Barbadoes General Hospital, by July 8th.

Hospital for Consumption and Diseases of the Chest, Brompton.—Resident House-Physician. Applications to the Secretary, by July 21st.

Leeds Union.—Assistant Medical Officer for the Workhouse Schools and Infirmary. Salary, £100 per annum, with board, washing, apartments, and attendance; unmarried, and not more than 35 years of age; doubly qualified. Applications on forms provided to be sent endorsed "Assistant Medical Officer," to John King, Clerk, Union Offices, East Parade, Leeds, by June 29th.

Newport and Monmouthshire Hospital. — House-Surgeon; doubly qualified. Salary, £100 per annum, with board and residence. no stimulants provided. Applications to the Secretary, by July 11th.

Royal Free Hospital, Gray's Inn Road, W.C.—House Surgeon; doubly qualified. Appointment for six months, but the holder is eligible for re-election for a further period of six months. No salary, but board, residence, and washing provided. Applications to C. W. Thies, Secretary, by June 27th.

Seamen's Hospital Society (Dreadnought), Greenwich, S.E.—House Surgeon for the Branch Hospital, Royal Albert and Victoria Docks, E. Salary £75 per annum, with board and residence; must be doubly qualified. Applications to P. Michelli, Secretary, by June 30th.

Taunton and Somerset Hospital.—House-Surgeon; doubly qualified. Must enter into an engagement for not less than three years in case the Governors shall so long require his services. Salary, £100 per annum. with board, lodging, and washing. Applications to J. H. Biddulph Pinchard, Secretary, 13, Hammet Street, Taunton, by July 13th.

University College, London.—Resident Medical Officer. Applications to J. M. Horsburgh, M.A., Secretary, by July 4th.

Western General Dispensary, Marylebone Road.—Second House-Surgeon; unmarried. Salary, £60 per annum, with board and residence. Applications to the Honorary Secretary, by June 29th.

Wolverhampton and Staffordshire General Hospital, Wolverhampton.—Resident Assistant. Appointment for six months. Board, lodging, and washing provided. Applications, inscribed "Applications for Resident Assistant," to be addressed to the Chairman of the Medical Committee by June 29th.

### Non-Resident Medical Officers.

County Borough of Huddersfield.—Medical Officer of Health. Salary, £400 per annum, with actual disbursements; must have a diploma in Sanitary Science. Applications, endorsed "Medical Officer of Health," to be sent to F. C. Lloyd, Town Clerk, Town Hall, Huddersfield, by July 7th.

Paddington Green Children's Hospital, W.—Ophthalmic Surgeon; must be F.R.C.S.Eng. Applications to the Secretary by July 11th.

West End Hospital for Diseases of the Nervous System, Paralysis, and Epilepsy, 73, Welbeck Street, W.—Physician; must be F. or M.R.C.P.Lond. Applications to B. Heckstall Smith, Secretary, by June 27th.

**Medical Appointment.**

SEAMEN'S HOSPITAL SOCIETY (Dreadnought).

WANTED, HOUSE SURGEON for Branch Hospital, Royal Victoria and Albert Docks, E.

Salary £75 per annum, with Board and Residence.

